# A Modified Surgical Technique for Steatocystoma Multiplex

**DOI:** 10.4103/0974-2077.63284

**Published:** 2010

**Authors:** Sanjiv Choudhary, Sankha Koley, Atul Salodkar

**Affiliations:** *Department of Dermatology, JNMC Sawangi, Wardha, Maharashtra, India*; 1 *Department of Dermatology, Bankura Sammilani Medical College, West Bengal, India*

**Keywords:** Radiofrequency, steatocystoma multiplex, surgery

## Abstract

**Background:**

Steatocystoma multiplex (SM) is a disorder of the pilosebaceous unit characterized by multiple sebum-containing dermal cysts. Different surgical modalities like cryosurgery, aspiration, surgical excision, incision with a surgical blade or sharp-tipped cautery followed by expression of cyst contents and forceps-assisted removal of the cyst wall and carbon dioxide laser have been used in the past.

**Aims:**

To study the efficacy of a modified surgical technique in the treatment of steatocystoma multiplex.

**Materials and Methods:**

We have used a simple modified surgical technique using a radiofrequency instrument as the incision tool for the treatment of SM in two patients.

**Results:**

The results were cosmetically excellent with no complications developing during or after the procedure. No recurrences were observed after five and half months of follow-up.

**Conclusions:**

This is a simple, easy, fast office-based procedure that is associated with minimal blood loss and post inflammatory hypo or hyperpigmentation and scarring are practically absent.

## INTRODUCTION

Steatocystoma multiplex (SM) is an uncommon disorder of the pilosebaceous unit characterized by the development of numerous sebum-containing dermal cysts. This condition may be hereditary (as an autosomal dominant trait) or more commonly sporadic. It usually begins in adolescence or early adult life. Clinically, it is characterized by multiple, small, soft, movable, yellowish-to-skin-colored dermal cystic papules and nodules,[1] varying from a few mm to 20 mm or more in size.[2] The overlying epidermis is usually normal with no central punctum.[2] Cysts tend to be asymptomatic, but often may be inflamed. The trunk and proximal extremities are commonly involved sites, but lesions may appear anywhere, including the scrotum.[3] The appearance of multiple cystic lesions involving the scrotum, especially in unmarried men, is highly embarrassing. Different surgical treatment options have been used in the treatment of SM. In this article, we describe a simple modified radiosurgical technique for the treatment of SM.

## CASE REPORTS

### Case 1

**Figure 1:**
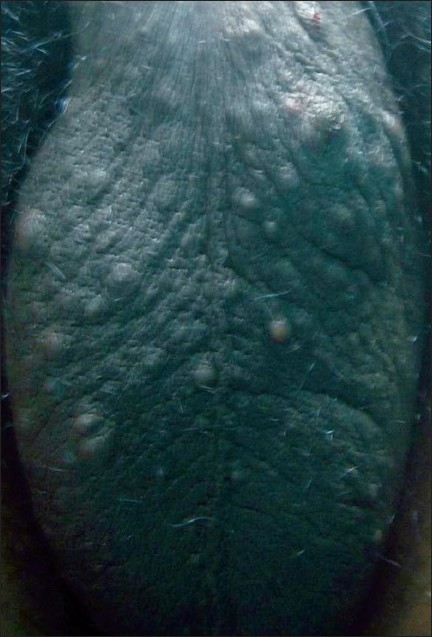
Asymptomatic skin colored papules and nodules on scrotum in Case 1

A 30-year-old male patient presented with multiple asymptomatic skin-colored papules and nodules that began around puberty involving the scrotum [Figure 1]. There was a family history of similar lesions being found in his father. The histopathological examination of a biopsy specimen showed the typical features of SM.

### Case 2

A 26-year-old male patient presented with multiple asymptomatic skin-colored and yellowish papules and nodules that began around puberty involving the scrotum. There was no family history of similar lesions. Histopathological findings of biopsy specimens were suggestive of SM.

## MATERIALS AND METHODS

**Figure 2:**
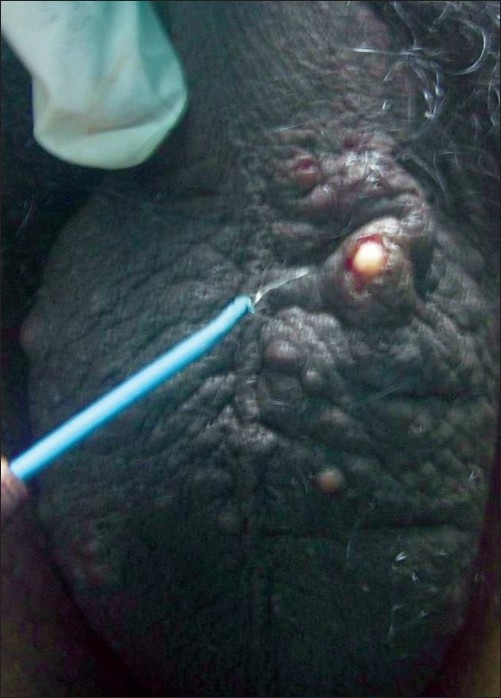
Mini-incisions are given on the cysts with the RF probe

**Figure 3:**
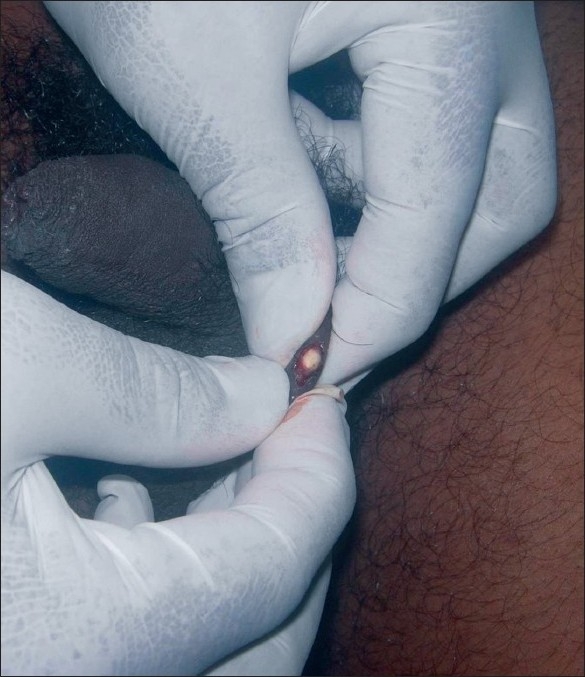
Cyst contents are squeezed out

**Figure 4:**
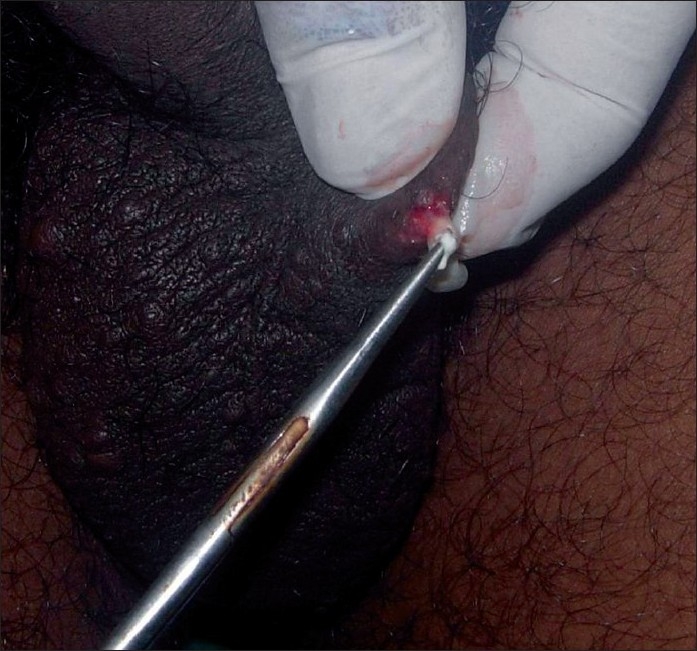
Extraction of the cyst by grasping the wall with forceps

**Figure 5:**
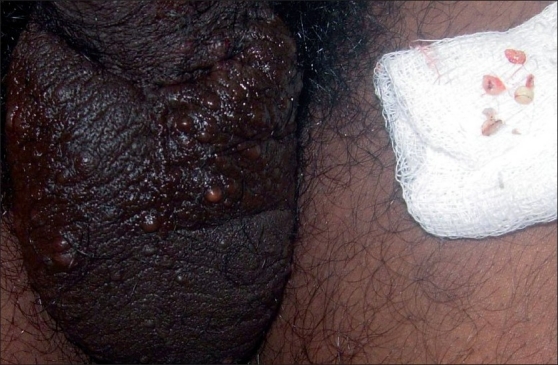
Extracted cysts placed on the gauze-piece

**Figure 6:**
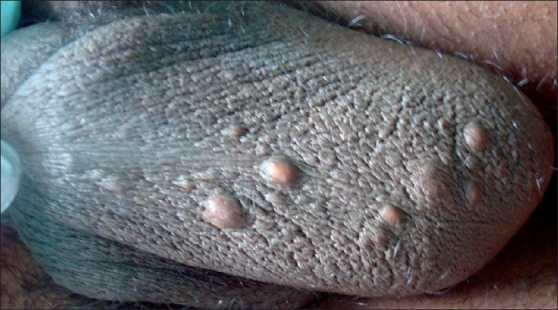
Case 2: Before procedure

**Figure 7:**
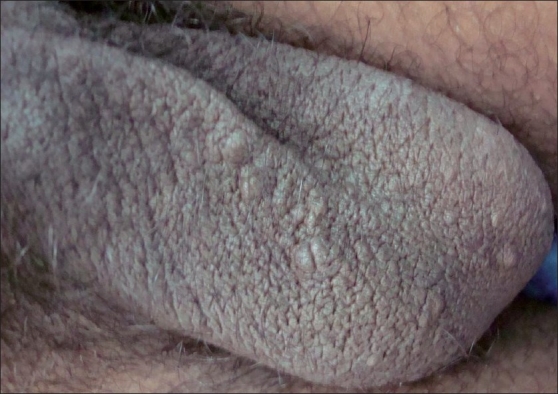
Case 2: After procedure

We have used a simple modified surgical technique using a radiofrequency (RF) instrument Bosco, power 2.4 MHz, as the incision tool for the treatment of SM in both the patients. Mini-incisions of 1-2 mm, following the relaxed skin tension lines (RSTL), were given on the cysts, under local anesthesia using the incision probe of the RF instrument [Figure 2]. The unipolar (monopolar) socket was used with the power output knob at the “medium” level. This was followed by expression of cyst contents by squeezing each cyst in between the thumb and the index finger [Figure 3]. Then, the cyst wall was grasped with the forceps and the sac was extracted through mini-incisions [Figures 4 and 5]. Stitches were not used as the incisions given were minimal and along the RSTL. More than 25 cysts were removed with this technique. All the lesions healed without any scar or postinflammatory hyperpigmentation [Figures 6 and 7]. The treatment was well tolerated by the patients. Preoperatively, all patients were given tetanus toxoid injection intramuscularly. After surgery, all of them were prescribed systemic antibiotics (a combination of cefadroxil 500 mg and clavulanic acid 125 mg) twice daily for 7 days, a painkiller twice daily for 5 days, and a topical antibiotic (nadifloxacin cream) twice daily for 10 days.

No complications developed during or after the procedure. After five and half months of follow-up, no recurrences were observed and the results were cosmetically excellent.

## DISCUSSION

SM is not an uncommon disorder in the Indian subcontinent. However, its treatment has not been discussed in details in the textbooks. Various surgical modalities have been used in the treatment of SM:

1. Cryosurgery: Cryosurgery has been used in the past but residual scarring is a limitation.[4]
2. Aspiration: Simple aspiration with an 18 gauge needle has been used successfully in minimizing scarring of facial lesions. Although postoperative scars are minimal, a high rate of recurrence has been observed. A variation of this method by insertion and gentle extirpation of cystic contents without removing the cyst wall is thought to be the treatment of choice in the management of facial lesions and those smaller than 1.5 cm in diameter.[5]
3. Surgical excision: Traditionally, surgery with elliptical excisions, flaps, or grafts was the most commonly practiced method. But often it is impractical for widespread lesions and has fallen out of favor due to its time-consuming nature and the associated risk of scarring. Punch excision followed by cyst removal has also been used in the past.
4. Incisional treatments: There are several type of incisional treatments. A number 11 surgical blade was used to produce a mini-incision followed by expression of cyst contents and excochleation of the cyst wall using a 1-mm curette. This resulted in minimal scarring and a low rate of recurrence.[6] A modified surgical technique, used on more than 50 lesions, is incision with a sharp-tipped cautery followed by expression of cyst contents and forceps-assisted removal of the cyst wall. This technique resulted in minimal depressed scarring and slight hypopigmentation with no evidence of recurrence.[7] A phlebectomy hook may also be used to produce small incisions, 2–3 mm in length, followed by a removal of the cyst wall.[8]
5. Carbon dioxide laser: The advent of carbon dioxide laser has allowed treatment of multiple lesions during a single treatment session, with no anesthesia, a low percentage of recurrence, and good cosmesis.[9]

In Case 1, SM was familial and in Case 2, it was sporadic. The inherited type of SM in the former was not associated with any nail disorder, keratoderma, or any feature of ectodermal dysplasia.

In our opinion, cryotherapy is associated with pain during the procedure, has got the risk of blister formation, and may be associated with postinflammatory hypo- or hyperpigmentation and scarring. The aspiration technique is not suitable for bigger and calcified cysts and also has the disadvantage of recurrence unless the cyst wall is not excised or ablated. With carbon dioxide laser, there are chances of scarring and postinflammatory hyperpigmentation while vaporizing the cyst wall. Also the therapy might not be cost effective. We have used the RF incision probe to make mini-incisions on the overlying skin. This technique has got the advantage of producing a bloodless field, which cannot be obtained with surgical blades. Multiple small nicks can be given in quick time while treating multiple cysts. In our experience, superficial cysts which are protruding over the skin surface can be easily removed. With RF probes, even the deep-seated cysts, which have their walls firmly adherent to the skin, can be dissected as well in a bloodless field. Charring of tissue, scarring, and postprocedure hyperpigmentation, noted with electrocautery, are practically absent with RF. The risk of recurrence is negligible as the cyst wall is completely removed with forceps.

This is therefore a simple, easy, office-based procedure that can be performed fast and also causes minimal blood loss.
